# Discovery of Superconductivity in Hard Hexagonal *ε*-NbN

**DOI:** 10.1038/srep22330

**Published:** 2016-02-29

**Authors:** Yongtao Zou, Xintong Qi, Cheng Zhang, Shuailing Ma, Wei Zhang, Ying Li, Ting Chen, Xuebing Wang, Zhiqiang Chen, David Welch, Pinwen Zhu, Bingbing Liu, Qiang Li, Tian Cui, Baosheng Li

**Affiliations:** 1 State Key Laboratory of Superhard Materials, College of Physics, Jilin University, Changchun, 130012, China; 2 Mineral Physics Institute, State University of New York, Stony Brook, N.Y. 11794, United States; 3 Department of Geosciences, State University of New York, Stony Brook, N.Y. 11794, United States; 4 Condensed Matter Physics and Materials Science Department, Brookhaven National Laboratory, Upton, N.Y. 11973, United States; 5 School of Science, Southwest University of Science and Technology, Mianyang, Sichuan 621010, China; 6 Department of Materials Science and Engineering, State University of New York, Stony Brook, N.Y. 11794, United States

## Abstract

Since the discovery of superconductivity in boron-doped diamond with a critical temperature (*T*_C_) near 4 K, great interest has been attracted in hard superconductors such as transition-metal nitrides and carbides. Here we report the new discovery of superconductivity in polycrystalline hexagonal *ε*-NbN synthesized at high pressure and high temperature. Direct magnetization and electrical resistivity measurements demonstrate that the superconductivity in bulk polycrystalline hexagonal *ε*-NbN is below ∼11.6 K, which is significantly higher than that for boron-doped diamond. The nature of superconductivity in hexagonal *ε-*NbN and the physical mechanism for the relatively lower *T*_*C*_ have been addressed by the weaker bonding in the Nb-N network, the co-planarity of Nb-N layer as well as its relatively weaker electron-phonon coupling, as compared with the cubic *δ*-NbN counterpart. Moreover, the newly discovered *ε*-NbN superconductor remains stable at pressures up to ∼20 GPa and is significantly harder than cubic *δ*-NbN; it is as hard as sapphire, ultra-incompressible and has a high shear rigidity of 201 GPa to rival hard/superhard material *γ*-B (∼227 GPa). This exploration opens a new class of highly desirable materials combining the outstanding mechanical/elastic properties with superconductivity, which may be particularly attractive for its technological and engineering applications in extreme environments.

Hard superconducting materials have attracted considerable interest in materials science, condensed matter physics and solid-state chemistry since the discovery of the superconductivity in superhard boron-doped diamond with a transition temperature of *T*_*C*_ ∼ 4 K^1^,^2^,^3^,^4^,^5^. For transition-metal nitrides, some of them possess very good superconductivity (*e.g.* 10 K for ZrN, 8.8 K for HfN, 17.3 K for *δ*-NbN), as well as excellent mechanical properties such as low compressibility, high shear rigidity and high hardness^6^,^7^,^8^,^9^,^10^,^11^,^12^,^13^,^14^,^15^,^16^. Despite the *T*_*C*_ for transition-metal nitrides is not very high, their remarkable mechanical properties make them good candidates of hard superconductors for specific electronic/high-field applications^17^ as well as potential applications in motor system, carbon-nanotube junctions and high-pressure devices^18^. In addition, the chemical inertness and high melting points also make these nitrides suitable for protective and wear-resistant coatings^19^.

It is known that rock-salt structured *δ*-NbN possesses the highest transition temperature among transition-metal nitrides (∼17 K), and has several polymorphs^4^,^8^,^20^,^21^,^22^,^23^ such as WC-type NbN, *δ*-NbN (NiAs-type) and hexagonal *ε*-NbN (#194, *P*6_3_/*mmc*), but only cubic *δ*-NbN has been extensively investigated^1^,^2^,^4^,^8^. Recent first-principles theoretical calculations of the thermodynamic properties and structural stability in NbN polymorphs^22^,^23^ (*e.g.* NaCl-, NiAs- and WC-type NbN) predicted that the hexagonal-structured NbN (*e.g.* WC- and NiAs-type) exhibited higher hardness and lower total energy than the cubic *δ*-NbN. These results indicated that the hexagonal phases were more stable than the cubic counterpart which appeared to be the most energetically unfavorable structure or metastable phase with the rock-salt structure.

For hexagonal *ε*-NbN polymorph, despite its crystal structure was ever simply referred by Terao^24^ and Holec *et al.*^25^, the experimental studies on hexagonal *ε*-NbN are very scarce, especially for its superconductivity and mechanical/elastic properties which have never been reported. Recently, the wide and growing interest lies in searching for novel materials with comprehensive superconductivity and excellent mechanical/elastic properties, which makes *ε*-NbN a good candidate of hard superconductors for the possible use in extreme environments. Here, we report the discovery of superconductivity in bulk polycrystalline hexagonal *ε*-NbN, and the findings of its ultra-incompressibility, high shear rigidity and hardness.

## Results

### Synthesis and structural stability of hexagonal *ε*-NbN at high pressure

**Figure 1:**
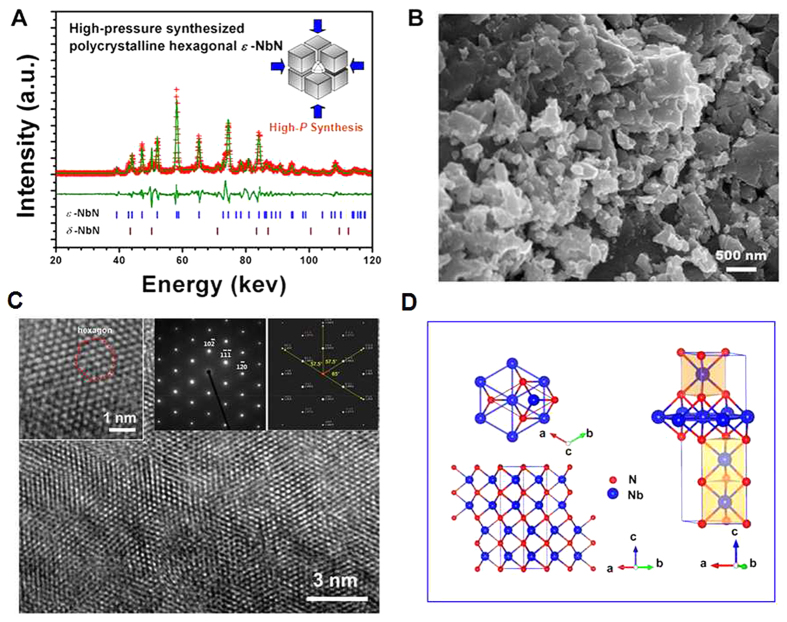
(**A**) Observed (red crosses) and fitted (olivine lines) synchrotron X-ray diffraction pattern of the synthesized bulk polycrystalline niobium nitride specimen for the present magnetization and electrical resistivity measurements. The peak positions of the hexagonal *ε*-NbN (PDF: #89-4757) and cubic *δ*-NbN (PDF: #74-1218) structures are denoted by tick marks. (**B**) SEM image showing the microstructure of the synthesized polycrystalline hexagonal-structured *ε*-NbN for the current measurements. The synthetic specimen was free of visible microcracks with an average grain size of about 1 *μ*m, exhibiting an equilibrated microstructure with homogeneous fine grains. (**C**) High resolution TEM (HRTEM) of the synthesized specimen; the corresponding observed and simulated SAED patterns and the enlarged portion of the HRTEM image are displayed as insets. (**D**) Crystal structure of the hexagonal *ε*-NbN (*P6*_*3*_*/mmc*, No. 194). The blue large and red small spheres represent Nb and N atoms, respectively.

Polycrystalline hexagonal *ε*-NbN bulk specimens used for the current magnetization and electrical resistivity measurements were synthesized from niobium nitride starting material (Goodfellow, claimed 99% purity) at 10 GPa and 1100∼1200 °C for 1.5 hour in a high-pressure multi-anvil apparatus at Stony Brook University. Details of this experimental setup were described elsewhere^26^,^27^,^28^,^29^,^30^,^31^. As shown in Fig. 1A, the synthetic specimen is almost a pure phase of *ε*-NbN with the hexagonal structure (PDF: #89-4757) coexisting with a minor amount of cubic *δ*-NbN. The volume fraction of cubic *δ*-NbN was estimated to be ∼2% from the intensity of the *δ*-NbN peaks observed in synchrotron X-ray diffraction; and there is not any other phases observed within the resolution of the current X-ray diffraction, such as tetragonal Nb_4_N_3_, hexagonal Nb_2_N, and so on.

Further SEM observations revealed that the synthesized specimen was free of visible microcracks with an average grain size of about 1-2 *μ*m, and exhibited an equilibrated and homogeneous microstructure, as shown in Fig. 1B. The corresponding composition analyses of the synthetic specimen yielded Nb_0.98(2)_N_0.96(5)_O_0.06(4)_ as determined by the SEM-EDX measurements, indicating that the as-synthesized specimen was almost oxygen-free *ε*-NbN or stoichiometric nitride within its uncertainty. The composite has also been studied by high-resolution TEM (HRTEM), which shows that the specimen possesses perfect crystalline form (Fig. 1C). The major phase was confirmed to be hexagonal *ε*-NbN. The up-mid inset to the HRTEM image shows the corresponding observed selected area electron diffraction (SAED) pattern along [211] axis, which can be indexed to the relevant reported structure (space group: *P6*_*3*_*/mmc*, No. 194). For further confirmation of the structure, the simulated SAED pattern projected along [211] axis is also shown in an up-right inset to Fig. 1C. The enlarged HRTEM, as an up-left inset in Fig. 1C, shows a clear hexagon formed by Nb atoms. Crystal structures of the transition-metal nitrides are generally characterized by strong intermetallic bonding with transition-metal atoms and N atoms occupying octahedral, tetrahedral or trigonal prismatic sites, giving rise to a large cohesive energy. In the hexagonal *ε*-NbN, each N atom is surrounded by six Nb atoms and there are six N atoms around the Nb atoms (Fig. 1D).

**Figure 2:**
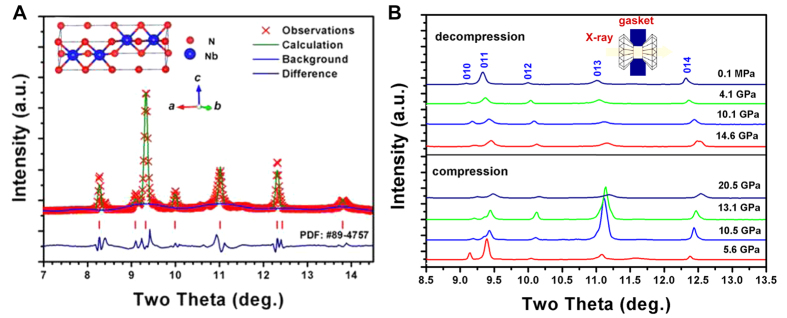
(**A**) Refined synchrotron angle-dispersive X-ray diffraction pattern of NbN powder at ambient conditions, suggesting a hexagonal-structured *ε*-NbN: (Space group: *P6*_*3*_*/mmc*, No. 194). Red crosses and olivine lines denote the observed and calculated profiles, respectively. The red tick marks correspond to the peak positions of hexagonal *ε-*NbN (PDF: #89-4757). The inset is the crystal structure of hexagonal *ε-*NbN. (**B**) Selected synchrotron angle-dispersive X-ray diffraction patterns of *ε*-NbN upon compression up to ∼20.5 GPa, in comparison with those during decompression where the peaks of the hexagonal phase were indexed (PDF: #89-4757).

The crystal structure of *ε*-NbN has been further explored by the refinement of high-resolution *in situ* angle-dispersive X-ray diffraction pattern (Fig. 2A), yielding *a* = 2.9599(4) Å, *c* = 11.2497(22) Å and *V*_*0*_ = 85.352(19) Å^3^ (*R*wp = 5.65%, *R*p = 2.60%, chi^2 = 0.5977) with a space group of *P6*_*3*_*/mmc* (No. 194). The lattice parameters are in good agreement with the previous experimental results (*a* = 2.960 Å, *c* = 11.270 Å) reported by Terao^24^, and comparable to our theoretically calculated results from GGA (*a* = 2.947 Å, *c* = 11.611 Å) and LDA (*a* = 2.908 Å, *c* = 11.464 Å), as well as the previous theoretical study (*a* = 2.993 Å, *c* = 11.415 Å)^25^. According to the well-known Born stability criteria^32^, the hexagonal *ε*-NbN is found to be mechanically stable, whereas the cubic *δ-*NbN is mechanically unstable based on our first-principles calculations of the elastic constants. Selected angle-dispersive synchrotron *in situ* X-ray diffraction patterns of *ε-*NbN upon compression in a diamond anvil cell (DAC) are shown in Fig. 2B. For comparison, the X-ray diffraction patterns of the specimen during decompression are also shown in Fig. 2B. Clearly, no phase transitions are observed throughout this experiment and the hexagonal-structured *ε*-NbN remains stable at pressure up to ∼20.5 GPa.

### Superconductivity in bulk polycrystalline hexagonal *ε*-NbN

**Figure 3:**
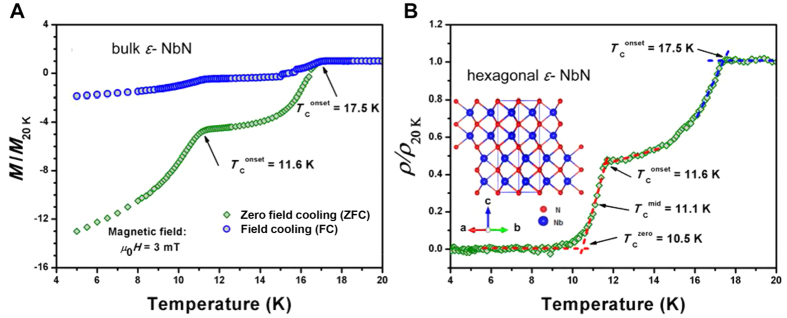
(**A**) Temperature dependence of the normalized magnetization *M*/*M*_20_ _K_ of bulk polycrystalline hexagonal *ε*-NbN at ambient pressure. (**B**) Electrical resistivity of the hexagonal *ε*-NbN specimen as a function of temperature at ambient pressure. The data are normalized to the values at 20 K (arrows show the superconducting transition points).

Figure 3A shows normalized magnetization for a bulk polycrystalline hexagonal *ε*-NbN as a function of temperature below 20 K under a magnetic field of 3 mT (or 30 Oe). Clearly, the magnetic susceptibility measurements reveal obvious diamagnetic responses at temperatures of ∼11.6 K and ∼17.5 K, respectively. The magnetic anomaly occurring at ∼17.5 K is ascribed to the superconducting transition of NaCl-structured cubic *δ*-NbN, while the magnetic anomaly at ∼11.6 K is related to the hexagonal *ε*-NbN phase. The existence of the hysteresis between the two magnetization curves for the zero-field cooling (ZFC) and field cooling (FC) modes shows that the hexagonal *ε*-NbN specimen is a typical type-II superconducting material.

The observed superconducting transition temperatures of *T*_*C*_ = 11.6 K and 17.5 K for the hexagonal ε-NbN and cubic *δ*-NbN, have been further addressed/confirmed by the current energy-dispersive X-ray diffraction pattern where the two-phase coexisting specimen is identified (Fig. 1A). As shown in Fig. 1A, the as-synthesized bulk polycrystalline hexagonal *ε*-NbN is coexisting with a minor amount of cubic *δ*-NbN. Using the Voigt bound for our theoretical calculations, we know that the abundance of *δ*-NbN of ∼2% will result in less than 1% difference in elastic moduli as compared with those for pure hexagonal *ε*-NbN^28^. This difference is within the experimental uncertainties, indicating that the effect of the minor *δ*-NbN on the elasticity of the nominal hexagonal-structured *ε*-NbN can be negligible. In contrast, the appearance of the minor *δ*-NbN (∼2%) has a significant effect on its electrical/magnetic properties. On the basis of the previous studies on the superconducting transition temperature^14^ (*T*_*C*_ ≈ 17.3 K) of *δ*-NbN and our XRD measurements, we thus conclude that the observed superconductivity at temperatures around 17.5 K should be attributed to minor phase of cubic *δ*-NbN, and the relatively low transition at 11.6 K is due to the hexagonal *ε*-NbN major phase. The nature of superconductivity in bulk hexagonal *ε*-NbN has further confirmed by our direct electrical resistivity measurements, exhibiting two resistive transitions at temperatures of ∼11.6 K and ∼17.5 K, respectively (Fig. 3B). These observed resistivity anomalies occur at almost the same temperatures with those obtained by magnetic susceptibility measurements (Fig. 3A). As shown in Fig. 3B, the onset transition temperature of *ε*-NbN is about 11.6 K, and zero resistivity is achieved at *T*_*C*_ = 10.5 K. By using the 90/10 criterion of superconducting transition temperature, we find that the midpoint of the electrical resistivity transition (*i.e.* the resistivity drops to 50% of that at 11.6 K) is about 11.1 K. The transition width is as small as ∼1 K, suggesting that the bulk polycrystalline hexagonal *ε*-NbN (nominal) specimen owns high quality and the homogeneous nature of the crystals.

### Anisotropic behavior and mechanical/elastic properties of hexagonal *ε*-NbN

**Figure 4:**
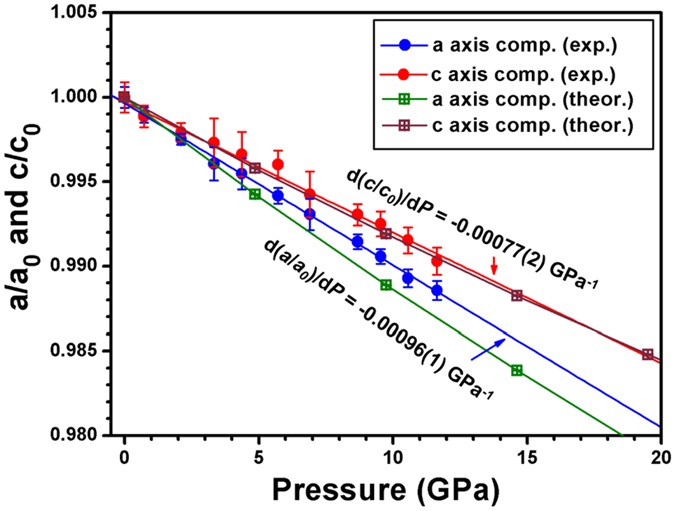
Experimental crystal-axis compression of hexagonal *ε*-NbN as a function of pressure, in comparison with the theoretical results by our first-principles calculations (GGA).

Niobium nitride polymorphs are attractive also due to their excellent mechanical/elastic properties (elastic behavior) besides their superconductivity, so understanding their hardness, elastic behavior, especially the Young’s modulus (*E*) and shear modulus (*G*), are of great importance for technological and engineering applications^4^. Compressibility measurements upon compression revealed a significant degree of anisotropy in the elastic behavior of hexagonal *ε*-NbN, where *ε*-NbN is more compressible along the *a*-axis direction, while stiffer along the *c*-axis. The least-squares fit of the lattice constants as a function of pressure yields d(*a/a*_*0*_)/d*P* = −0.00096(1) GPa^−1^ and d(*c/c*_*0*_)/d*P* = −0.00077(2) GPa^−1^, as shown in Fig. 4. For comparison, our theoretical first-principles calculations of the pressure-dependent lattice constants are also displayed here, agreeing well with our experimental data, especially for the compressibility of *c*-axis.

**Figure 5:**
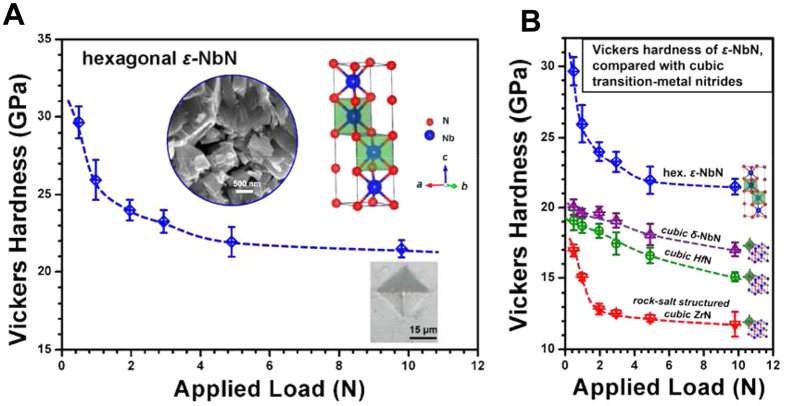
(**A**) Average measured Vickers hardness (*H*_*V*_) for polycrystalline hexagonal *ε*-NbN under different applied loads, indicating that the tendency of hardness decreases and becomes weak with large loads; SEM image, crystal structure and a typical Vickers indentation image at a load of 9.8 N are displayed as insets. (**B**) Comparing the Vickers hardness of hexagonal *ε*-NbN with those for the rock-salt structured cubic NbN, HfN and ZrN reported by Chen *et al.* (ref. 4).

Hardness measurements were performed on the synthesized polycrystalline hexagonal *ε*-NbN by means of the Vickers indentation method using a pyramidal diamond indenter. The loading force of the hardness tester is adjusted from 2.94 to 9.8 N (0.49, 0.98, 1.98, 2.94, 4.9 and 9.8 N loads). The dwelling time was fixed at 15 s. At each applied load, five indentations were performed. Under a certain applied load of *P*, the hardness (*H*_*V*_) was determined by *H*_*V*_ = 1854.4*P*/*d*^2^, where *d* is the arithmetic mean of the two diagonals of the indent in micrometers^33^,^34^. The average *H*_*V*_ values were 29.7 ± 1.0, 26.0 ± 1.3, 24.0 ± 0.7, 21.9 ± 1.0 and 21.5 ± 0.6 GPa under a load of 0.49, 0.98, 1.98, 2.94, 4.9 and 9.8 N, respectively. The results show that the hardness appears to increase with a decrease under various loading forces. The average measured Vickers hardness *H*_*V*_ for polycrystalline hexagonal *ε*-NbN under different loads are shown in Fig. 5A. Clearly, the hexagonal *ε*-NbN exhibits a Vickers hardness of 22∼30 GPa, which is significantly harder than the high-pressure synthesized polycrystalline ReB_2_ (17∼19 GPa) as reported by Qin *et al.*^34^ and is almost as hard as sapphire Al_2_O_3_^4^. Figure 5B shows a summary of the Vickers hardness of *ε*-NbN as a function of applied load, compared with the previous study on rock-salt structured cubic NbN, HfN and ZrN, showing that the hexagonal-structured *ε*-NbN is consistently harder than cubic NbN (17∼20 GPa), HfN (15.5∼19.1 GPa) and ZrN (11.8∼16.9 GPa) under various loading forces ranging from 0 to 9.8 N, respectively^4^.

It is widely accepted that bulk and/or shear moduli can reflect the hardness in an indirect way^27^,^35^. To further explore the correlations between the elastic modulus (*B*, *G*, *E*) and other physical properties, we have performed *in situ* ultrasonic measurements on hexagonal *ε*-NbN at high pressure. The experimental procedure in details can be seen elsewhere^26^,^27^,^28^,^29^,^30^,^31^. The high-pressure elasticity and sound velocities of hexagonal *ε*-NbN are out of the current scope of this paper and will be published elsewhere^28^. The ambient-condition bulk and shear moduli derived from the acoustic measurements on *ε*-NbN yielded *B*_*S0*_ = 373(2) GPa and *G*_*0*_ = 201(1) GPa^28^. Clearly, hexagonal *ε*-NbN exhibits a remarkable incompressibility, which is as incompressible/stiff as superhard material *c*BN (∼381 GPa)^36^. The shear rigidity of *ε*-NbN (∼201(1) GPa) rivals that for superhard *γ*-B (∼227 GPa)^37^, which is well consistent with the theoretical shear modulus/rigidity of *G*_*0*_ = 199 GPa by our first-principles calculations. According to our experimentally obtained bulk (*B*_*S*_) and shear (*G*) moduli, the Young’s modulus (*E*) is derived to be 510(1) GPa by applying the equation *E = *9*B*_*S*_*G/(*3*B*_*S*_* + G)*, which surpasses that of superhard B_6_O-B_4_C composite (501 GPa)^38^, and can be comparable to that of polycrystalline *c*BN (587 GPa)^39^, indicating that the hexagonal *ε*-NbN will also be a good candidate for mechanical applications.

## Discussion

### Mechanism of superconductivity in hexagonal *ε*-NbN

**Figure 6:**
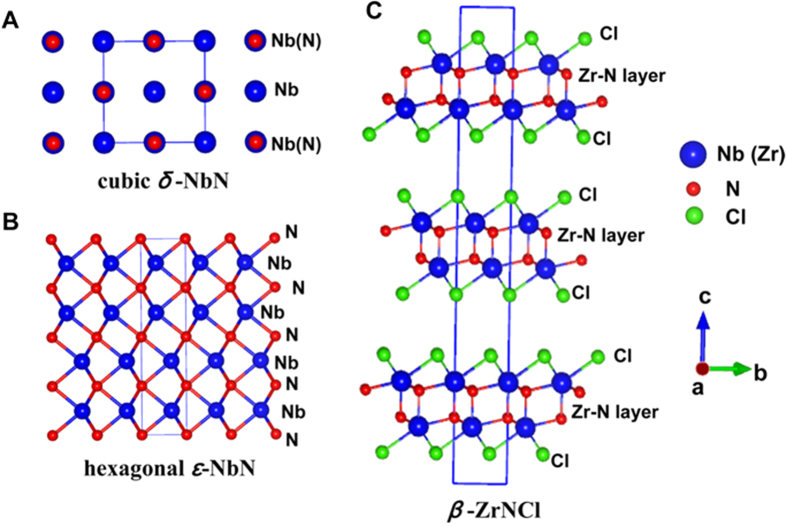
Comparison of the atomic structures of cubic *δ*-NbN **(A),** hexagonal *ε*-NbN (**B**) and rhombohedral *β*-ZrNCl (**C**) along *a* axis. The corresponding single unit cell is displayed in solid line. Blue, red, and green spheres stand for Zr/Nb, N and Cl atoms, respectively.

It is well known that the rock-salt structured transition-metal nitrides (*e.g.* ZrN, NbN, HfN) and the rhombohedral *β*-ZrNCl (∼13 K) and *β*-HfNCl (∼26 K) compounds show good superconductivity^8^,^15^,^40^,^41^. The structures of cubic *δ*-NbN, hexagonal *ε*-NbN and rhombohedral *β*-ZrNCl along *a* axis are shown in Fig. 6. In the hexagonal-structured *ε*-NbN, each N atom is surrounded by six Nb atoms and there are six N atoms around the Nb atoms (Fig. 6B). The rhombohedral *β*-ZrNCl can be considered to be composed of alternate stacking of honeycomb ZrN bilayers sliced from a ZrN crystal of the hexagonal structure and sandwiched by chloride layers, as shown in Fig. 6B,C. Therefore, the Nb-N layer in hexagonal-structured *ε*-NbN, as shown in Fig. 6B, is considered a critical component to stabilizing its superconductivity.

To gain insight into the mechanism of superconductivity in hexagonal *ε*-NbN against those for the rock-salt structured nitrides as well as rhombohedral *β*-ZrNCl, their crystal structures (Fig. 6) and their structural parameters have been further investigated. The lattice constants, average bond length, bond angle and superconducting transition temperatures (*T*_*C*_) of niobium nitrides derived from our first-principles calculations and magnetic/electrical measurements are summarized in Table 1, in comparison with those of cubic ZrN and *β*-ZrNCl superconductors^40^,^41^. Our theoretical calculations show that the average bond length of Nb-N for hexagonal *ε*-NbN (2.2219 Å) is longer than those of the NaCl-structured NbN (2.2077 Å) and rhombohedral *β*-ZrNCl (2.2127 Å), but shorter than that of cubic ZrN (2.2890 Å). As shown in Fig. 6, the average bond angle (N-Nb-N) for the distorted NbN_6_ (trigonal prismatic coordination) of *ε*-NbN is ∼82.25°, and the layered hexagonal-structured *ε-*NbN is almost coplanar against the NaCl-structured NbN/ZrN with the bond angle of 90°. Therefore, the weaker bonding in the Nb-N network and the co-planarity may be the reason for the relatively lower *T*_*C*_ (∼11.6 K) compared with the cubic *δ*-NbN counterpart (∼17.5 K). This correlation is further supported by the rock-salt structured ZrN and rhombohedral *β*-ZrNCl superconductors (Table 1), where a stronger bonding of Zr-N in *β-*ZrNCl (2.2127 Å) results in a higher *T*_*C*_ (∼13.0 K), in comparison with the NaCl-structured ZrN (2.2890 Å, 10.7 K). On the other hand, the shorter average bond length of N-N (2.9728 Å) for *ε*-NbN together with the lower total energy, compared with *δ*-NbN (N-N: 3.2122 Å), indicated that the hexagonal *ε*-NbN is more stable than the cubic counterpart.

For phonon-mediated superconductivity, *T*_*C*_ is given by McMillan’s formula 
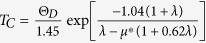
, where *Θ*_*D*_ is the Debye temperature, μ* is the Coulomb pseudopotential or electron-electron interaction constant and *λ* is the electron-phonon coupling^42^. Based on the obtained elastic bulk and shear moduli together with the ambient-condition density *ρ* = 8.30(2) g/cm^3^ of hexagonal *ε*-NbN as derived from our ultrasonic measurements^28^, the Debye temperature *Θ*_*D*_is determined to be ∼738 K from the equation^28^ described as 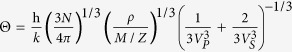, which is significantly larger than the theoretical result of *Θ*_*D*_ = 629 K for cubic *δ-*NbN^43^, and is double the previously experimental value of ∼363 K (ref. 11). Using the obtained values of *Θ*_*D*_ = 738 K, *T*_*C*_ = 11.6 K, and a well-accepted Coulomb parameter μ* = 0.1∼0.13 for transition-metal nitrides^3^, we estimate the electron-phonon coupling constant *λ* = 0.57∼0.63. This yielded electron-phonon coupling *λ* = 0.57∼0.63 for *ε*-NbN is significantly weaker than those (*λ* = 0.87 and 0.906) for cubic *δ-*NbN counterpart 8,9.

It is clearly seen from the McMillan’s formula^42^ that the effects of *λ* (as the exponential term) on the *T*_*C*_ is much more significant than *Θ*_*D*_ (as the linear term), which can shed light on the relatively smaller *T*_*C*_ = 11.6 K, compared with *T*_*C*_ = 17 K for *δ-*NbN^44^. The relatively small value of *λ* for *ε*-NbN can be understood qualitatively from the relation of 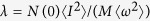, where *N*(0) is the density of electronic states at the Fermi energy, <*I*^*2*^> is the average square of the electron-phonon matrix element, *M* is the ionic mass and <*ω*^*2*^> is a characteristic phonon frequency averaged over the phonon spectrum having 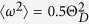 (ref. 42). Although the ionic mass of *ε*-NbN is similar to that of *δ-*NbN, its stronger covalent bonds in *ε*-NbN imply a smaller *N*(0) and larger <*ω*^*2*^>, compared to those for *δ-*NbN. These results have been further confirmed by experimentally measured *Θ*_*D*_ = 738 K for *ε*-NbN, which is larger than the *Θ*_*D*_* = *629 K for *δ-*NbN^43^.

First-principles calculations show that the superconducting and mechanical properties for transition-metal nitrides/carbonitrides are closely related to their electronic properties^45^,^46^. For a good understanding of the mechanical/superconducting properties of *ε*-NbN, electronic properties of the total densities of states (TDOS) and partial densities of states (PDOS) for hexagonal *ε*-NbN at ambient pressure have been calculated, in comparison with those for cubic *δ*-NbN counterpart (see Supplementary Materials: Fig. S1). Both hexagonal *ε*-NbN and cubic *δ*-NbN show similar metallic bonding features with a finite DOS at the Fermi level (*E*_*F*_), originating mostly from the 4*d* electrons of Nb and 2*p* electrons of N and agree well with our electrical measurements that *ε*-NbN is a metallic electrical conductor at ambient conditions. Clearly, there is a strong hybridization between Nb 4*d* and N 2*p* states in *ε-*NbN as revealed by the appearance of “pseudogap” just below and/or above the Fermi level, indicating the covalent and/or ionic bonding between Nb and N atoms (Fig. S1(A)). When comparing with the electronic structures of hexagonal *ε*-NbN and cubic *δ*-NbN, we note that there is a small peak dominated by the Nb-*d* orbital at about −0.68 eV in the DOS for *ε*-NbN (Fig. S1(A,B)). The appearance of this peak with low energy indicates a stronger bonding arising from the metal *d* orbitals in *ε*-NbN as compared to *δ*-NbN, resulting in an enhancement of the elastic/mechanical strength of *ε*-NbN. Fig. S1(A) shows that the TDOS around the Fermi level (*E*_*F*_) lies in a dip for *ε*-NbN, whereas the density of states increases monotonically at *E*_*F*_ for *δ*-NbN (Fig. S1(B)). This agrees well with the result from the total-energy calculations that the hexagonal *ε*-NbN is more stable than the cubic counterpart.

As reported by Oya *et al.*^47^, tetragonal phases *γ*-Nb_4_N_3_ and Nb_4_N_5_ with long-range-ordered arrangement of vacancies exhibited superconductivity^47^, whereas the hexagonal NbN and Nb_5_N_6_ didn’t show superconductivity at temperatures down to 1.77 K. For the mechanism of superconductivity in transition-metal nitrides, it is suggested that the continuous promotion of *s*, *p* electrons to the *d* shell in all solids under pressure is one of the factors which will induce superconductivity. As seen from Fig. S1, the contribution of the 4*d-*state is larger than those of the 5*s* and 5*p* states. The larger contribution of 4*d* state electrons clearly shows the possibility of superconductivity in hexagonal-structured NbN at ambient pressure.

In summary, we have discovered the superconductivity at ∼11.6 K in bulk polycrystalline hexagonal *ε*-NbN, which was synthesized at high pressure and high temperature in a high-pressure multi-anvil apparatus. The weaker bonding in the Nb-N network and the co-planarity may be the reason for the relatively lower *T*_*C*_ (∼11.6 K) compared with the cubic *δ*-NbN counterpart (∼17.5 K). Our theoretical calculations reveal that the contribution of the 4*d-*state is larger than those of the 5*s* and 5*p* states, and the relatively larger contribution of 4*d* state electrons may be responsible for the superconductivity in hexagonal *ε*-NbN. In addition, the hexagonal *ε*-NbN was found to exhibit excellent mechanical properties, which is as hard as sapphire Al_2_O_3_ (21∼23 GPa)^4^ and possessed a remarkable incompressibility^36^ (as stiff as superhard *c*BN of ∼381 GPa). The shear rigidity of *ε*-NbN (∼201(1) GPa) rivals that for superhard *γ*-B (∼227 GPa)^37^, and the Young’s modulus (∼510(1) GPa) is surpassing that for B_6_O-B_4_C composite (501 GPa)^38^. Our theoretical calculations indicate that the hexagonal *ε*-NbN is more stable than the cubic *δ*-NbN, and the stronger bonding arising from the metal *d* orbitals in *ε*-NbN compared to *δ*-NbN results in an enhancement of the elastic/mechanical strength of *ε*-NbN. This study opens a new window for the design of desirable materials with the combination of excellent mechanical properties and superconductivity, which may be particularly attractive for its technological and engineering applications in extreme conditions.

## Methods

### Magnetization and electrical resistivity measurements on polycrystalline hexagonal *ε-*NbN

Magnetization measurements of the high-pressure synthesized bulk polycrystalline hexagonal *ε-*NbN were performed in a Superconducting Quantum Interference Device (SQUID) based magnetometer (MPMS, Quantum Design). Electrical resistivity measurements on hexagonal *ε-*NbN were conducted in a Physical Property Measurement System (PPMS, Quantum Design) using the standard four-probe method^48^.

### *In situ* X-ray diffraction study of hexagonal ε-NbN at high pressure

High-pressure synchrotron X-ray experiments using diamond-anvil cell (DAC) techniques were performed at the X17C beamline of National Synchrotron Light Source, Brookhaven National Laboratory. Stainless T301 steel plates with an initial thickness of 250 μm were used as gaskets. The *ε*-NbN powder, a tiny ruby ball, and the methanol-ethanol pressure medium (4:1) were loaded into the hole in the gasket. The experimental cell-pressure was determined by the pressure-induced fluorescence shift of ruby^49^. The incident synchrotron radiation beam was monochromatized to a wavelength of 0.40722 Å. The collected two-dimensional X-ray diffraction patterns were analyzed by integrating 2D images as a function of *2θ* using the program Fit2D to obtain conventional, one-dimensional profiles^50^.

### First-principles calculations

Our first-principles calculations were performed with the CASTEP code^51^, based on density functional theory (DFT) using Vanderbilt-type ultrasoft pseudopotentials and a plane-wave expansion of the wave functions^52^. The local density approximation (LDA) and generalized gradient approximation (GGA) in the scheme of Perdew-Burke-Ernzerhof (PBE) were employed for determination of the exchange and correlation potentials for electron-electron interactions. The Broyden-Fletcher-Goldfarb-Shanno optimization method was applied to search for the ground states of *ε*-NbN. For the Brillouin-zone sampling, the Monkhorst-Pack scheme was adopted^53^. To confirm the convergence of our calculations, we have carefully analyzed the dependences of the total energy on the cutoff energy and the *k*-point set mesh according to the Monkhorst-Pack grid. During our first-principles calculations, the difference in total energy was minimized to below 5 × 10^−7^ eV/atom, the maximum ionic Hellmann-Feynman force is converged to less than 0.01 eV/Å, and the total stress tensor is reduced to the order of 0.02 GPa by using the finite basis-set corrections. The valance configuration is *4p*^*6*^*5 s*^*1*^*4d*^4^ *and* 2*s*^2^2*p*^3^ for Nb and N, respectively. Integrations in the Brillouin zone are performed using special k points generated with 10 × 10 × 2. One-electron valence states are expanded on a basis of plane waves with a cutoff energy of 600 eV in the electronic property calculations. All these parameters have been tested to be sufficient for the convergence.

## Additional Information

**How to cite this article**: Zou, Y. *et al.* Discovery of Superconductivity in Hard Hexagonal *ε*-NbN. *Sci. Rep.* **6**, 22330; doi: 10.1038/srep22330 (2016).

## Supplementary Material

Supplementary Information

## Figures and Tables

**Table 1:** Structural parameters and superconducting transition temperatures (*T*_*C*_) of transition-metal nitrides.

Compounds	hexagonal *ε*-NbN	*δ*-NbN	cubic ZrN	*β-*ZrNCl
Lattice constant (Å)				
* a*	2.9722	4.4154	4.576	3.606
* c*	11.2891	4.4154	4.576	27.67
Bond length (Å)				
* *M-N	2.2219	2.2077	2.2890	2.2127
* *N-N	2.9728	3.2122	–	–
Bond angle				
* *N-M-N	82.3°	90°	90°	115.9°
* T*_*C*_(K)	11.6	17.5	10.7	13.0
* *Ref.	This study	This study	Ref. 40	Ref. 40,41
