# Supplementary material for: Discovery of Superconductivity in Hard Hexagonal ε-NbN
Source: Sci Rep. 2016 Feb 29;6:22330. doi: 10.1038/srep22330 (PMC4770320; doi:10.1038/srep22330)
Supplement: Supplementary Information [file srep22330-s1.pdf]

Discovery of Superconductivity in Hard Hexagonal ε-NbN

Yongtao Zou[1,2], Xintong Qi[3], Cheng Zhang[4], Shuailing Ma[1], Wei Zhang[5], Ying Li[2], Ting Chen[3], Xuebing Wang[3], Zhiqiang Chen[2], David Welch[4,6], Pinwen Zhu[1], Bingbing Liu[1], Qiang Li[4], Tian Cui[1] & Baosheng Li[2]

[1]State Key Laboratory of Superhard Materials, College of Physics, Jilin University, Changchun, 130012, China.

[2]Mineral Physics Institute, State University of New York, Stony Brook, N.Y. 11794, United States.

[3]Department of Geosciences, State University of New York, Stony Brook, N.Y. 11794, United States.

[4]Condensed Matter Physics and Materials Science Department, Brookhaven National Laboratory, Upton, N.Y. 11973, United States.

[5]School of Science, Southwest University of Science and Technology, Mianyang, Sichuan 621010, China.

[6]Department of Materials Science and Engineering, State University of New York, Stony Brook, N.Y. 11794, United States.

*Correspondence and requests for materials should be addressed to Y. Z. (E-mail: yongtaozou@jlu.edu.cn or yongtaozou8@yahoo.com);

**The current ultrasonic, magnetization and electrical resistivity measurements were performed in the U. S. when Yongtao Zou was working at the State University of New York (Stony Brook).

Supplementary Materials

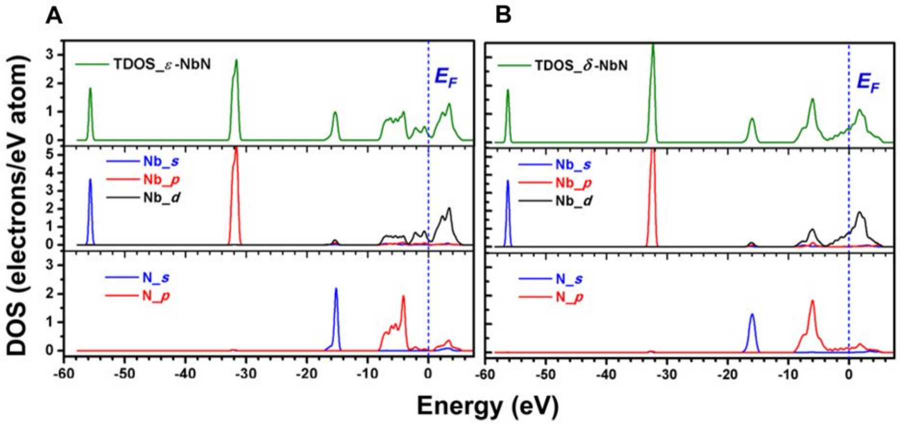

Figure S1. (A). Total and partial density of states for hexagonal ε-NbN at ambient pressure,

in comparison with those for rock-salt structured cubic δ-NbN (B). Reproduced with permission from Zou *et al*., (2015). Copyright 2015 Nature Publishing Group.

References

1. Zou, Y., Wang, X., Chen, T., Li, X., Qi, X., Welch, D., Zhu, P., Liu, B., Cui, T. & Li, B. Hexagonal-structured ε-NbN: ultra-incompressibility, high shear rigidity, and a possible hard superconducting material. *Sci. Rep.* **5***,* 10811 (2015).
